# The effect of auditory verbal imagery on signal detection in hallucination-prone individuals

**DOI:** 10.1016/j.cognition.2015.09.015

**Published:** 2016-01

**Authors:** Peter Moseley, David Smailes, Amanda Ellison, Charles Fernyhough

**Affiliations:** a Psychology Department, Durham University, South Road, Durham DH1 3LE, UK; b School of Psychology, University of Central Lancashire, Preston PR1 2HE, UK; c Department of Psychology, Leeds Trinity University, Horsforth, Leeds, LS18 5HD, UK

**Keywords:** Auditory imagery, Inner speech, Hallucinations, Imagery–perception interaction

## Abstract

Cognitive models have suggested that auditory hallucinations occur when internal mental events, such as inner speech or auditory verbal imagery (AVI), are misattributed to an external source. This has been supported by numerous studies indicating that individuals who experience hallucinations tend to perform in a biased manner on tasks that require them to distinguish self-generated from non-self-generated perceptions. However, these tasks have typically been of limited relevance to inner speech models of hallucinations, because they have not manipulated the AVI that participants used during the task. Here, a new paradigm was employed to investigate the interaction between imagery and perception, in which a healthy, non-clinical sample of participants were instructed to use AVI whilst completing an auditory signal detection task. It was hypothesized that AVI-usage would cause participants to perform in a biased manner, therefore falsely detecting more voices in bursts of noise. In Experiment 1, when cued to generate AVI, highly hallucination-prone participants showed a lower response bias than when performing a standard signal detection task, being more willing to report the presence of a voice in the noise. Participants not prone to hallucinations performed no differently between the two conditions. In Experiment 2, participants were not specifically instructed to use AVI, but retrospectively reported how often they engaged in AVI during the task. Highly hallucination-prone participants who retrospectively reported using imagery showed a lower response bias than did participants with lower proneness who also reported using AVI. Results are discussed in relation to prominent inner speech models of hallucinations.

## Highlights

• Investigated relation between hallucinations, mental imagery and signal detection.
• Individuals prone to hallucinations showed a lower SDT response bias with imagery.
• Finding held for both instructed and self-reported use of auditory verbal imagery.
• Atypical auditory imagery may lead to the generation of auditory hallucinations.

## 1 Introduction

### 1.1 Auditory verbal hallucinations and inner speech

Auditory verbal hallucinations (AVHs) are the experience of hearing a voice in the absence of any speaker. Although commonly associated with a diagnosis of schizophrenia, AVHs also occur in around 1.5–3% of the healthy, nonclinical population (Tien, 1991). There is emerging evidence that the predisposition to AVHs may lie on a continuum, ranging from individuals who frequently experience, to individuals who rarely or never report, hallucinations (Johns and van Os, 2001, Johns et al., 2014). A fruitful area of investigation is therefore to investigate whether cognitive traits and biases associated with hallucinations in clinical populations are shared by individuals in the general population who report frequent hallucinatory experiences (Badcock & Hugdahl, 2012).

The most prominent cognitive model of AVHs suggests that they occur when an internal mental event (such as inner speech or auditory verbal imagery – AVI) is misattributed to an external source (Ditman and Kuperberg, 2005, Frith, 1992, Jones and Fernyhough, 2007b). This strand of research has been embedded in the source monitoring framework, which attempts to explain how we make judgements regarding the origin of information (i.e., its source; Johnson, Hashtroudi, & Lindsay, 1993). Specifically, an externalising bias in reality monitoring, which refers to the ability to distinguish between internally generated and externally generated perceptions, has been linked to AVHs (Bentall, Baker, & Havers, 1991). Externalising biases have variously been linked to excessively vivid mental imagery (Aleman, Böcker, Hijman, de Haan, & Kahn, 2003), and low cognitive effort/intrusiveness associated with mental imagery (Jones and Fernyhough, 2009, Morrison et al., 1995). On a mechanistic level, forward models may be involved in predicting the sensory consequences of motor processes, and successful prediction via an efference copy may be one way in which self-generated actions are experienced as such (Frith, Blakemore, & Wolpert, 2000). Aberrant efference copy mechanisms could therefore underlie the misattribution of internal mental events to an external, non-self source (Ford & Mathalon, 2005).

Reality monitoring for verbal stimuli has typically been assessed using source memory paradigms, which require participants to recall whether words were spoken by the experimenter or by themselves. A common finding is that patients with a diagnosis of schizophrenia who hallucinate, compared to those who do not hallucinate, are more likely to misremember words as having been spoken by the experimenter, but do not make the reverse error (e.g., Brunelin et al., 2006, Woodward et al., 2007). That is, participants who hallucinate tend to show an ‘externalising bias’ on reality monitoring tasks. Consistent with continuum models of AVHs (Van Os, Hanssen, Bijl, & Ravelli, 2000), non-clinical samples who report higher levels of hallucination-proneness also show a similar pattern of responding on reality monitoring tasks (Brookwell et al., 2013, Larøi et al., 2004).^1^

However, these tasks are not ideally positioned to test models of AVHs that specify the misattribution of internal mental events such as inner speech, for two main reasons: (1) they are not ‘online’ measures (source memory tasks, for example, are ‘offline’ in that they require participants to decide who generated words earlier in the testing session); (2) they are either not specific to monitoring of speech or, if they are, are likely to use ‘overt’ (out loud) speech, as opposed to engaging the participant in auditory verbal imagery or inner speech. This limits the applicability of the results to inner speech models of AVHs, because it assumes that overt vocalisation in an experimental situation utilises the same mechanisms as covert or inner speech. Although there is evidence that overt and covert speech share cognitive and neural mechanisms, particularly in relation to the motor system (Alderson-Day and Fernyhough, 2015, Perrone-Bertolotti et al., 2014), any inferences from studies using overt speech about the nature of covert speech are necessarily indirect, and based on the assumption that similarities between the two are more significant than the differences.

A similar line of research has attempted to engage the participant in an ‘online’ decision making process, referred to as ‘reality discrimination’, requiring participants to immediately respond as to whether a perception was internal or external (in contrast to the ‘offline’ decisions required in a source memory task, which typically require a decision to be made at a later time point, e.g., Woodward et al., 2007). Reality discrimination tasks typically take the form of signal detection tasks, in which the participant must decide whether a voice was present in a burst of noise. In these tasks, hallucinating patients tend to show a bias towards responding that a voice is present in the noise (Varese, Barkus, & Bentall, 2012). In a non-clinical sample, participants who reported more hallucinatory experiences also showed the same bias in responding (Barkus et al., 2011). These findings have been linked theoretically to the reality monitoring tasks described above, as providing evidence linking AVHs to an externalising bias (Brookwell et al., 2013). It is unclear, however, to what extent performance on auditory signal detection tasks relates to inner speech/AVI processes, since participants are not specifically instructed to use imagery during the task. One way to address this concern would be to employ a paradigm that requires participants to engage in covert AVI, whilst simultaneously detecting the presence or absence of a similar auditory verbal stimulus.

### 1.2 Mental imagery and perception

Previous research on the interaction between mental imagery and perception has come closest to meeting the two criteria outlined above (engaging participants in an online task, and controlling the mental imagery they generate while performing the task). Perky (1910) carried out a series of experiments that suggested that visual imagery interfered with the simultaneous perception of a visually presented stimulus (subsequently referred to as the Perky Effect). For example, participants who engaged in visual mental imagery of an object took longer to detect a visually presented stimulus of the same object than did participants who did not generate any mental imagery. This was taken to indicate that, since mental imagery and perception could be confused, they must rely on similar mechanisms.

However, others have found that mental imagery actually facilitates perception in the visual modality (Peterson & Graham, 1974). This finding has also been replicated in the auditory modality; for example, Farah and Smith (1983) engaged participants in auditory imagery of a pure tone, whilst simultaneously requiring them to detect a similar tone in noise. Participants were therefore required to distinguish between self-generated, internal mental imagery and an external stimulus. Using auditory imagery facilitated perception of the tone, although the task used did not include trials with no signal present, and so signal detection analysis was not reported. Findings on the interaction between imagery and perception have, therefore, been equivocal. More recently, Aleman et al. (2003) used a similar paradigm with a sample of patients with a diagnosis of schizophrenia, showing that the ‘gain’ on perception of a pure tone due to auditory imagery was strongly correlated with hallucination severity. This finding was interpreted as reflecting an over-reliance on top-down processes in hallucinating patients (which could also be related to a bias towards labelling internal imagery as external).

One problem with these studies is that they do not measure the effect of imagery on the tendency to *falsely* detect a signal in noise,^2^ because there is always a signal present. This is a key variable when linking performance to the tendency to hallucinate, and also when performing signal detection analysis. From the data presented by Aleman et al. (2003), for example, it is not possible to tell whether the ‘gain’ on perception was due to a change in sensitivity (an increased ability to distinguish signal from noise), or a change in response bias (i.e., participants being more willing to respond that a tone was present when using imagery). The previously discussed literature relating to biases in reality monitoring/discrimination would imply that it may be the latter. Imagery–perception interaction tasks, though, have the advantage of directly engaging participants in internal mental imagery (as opposed to speaking aloud), and requiring them to distinguish whether any subsequent perception was internally generated or not, hence addressing the concern about reality discrimination tasks described above. As well as being informative on the nature of mental imagery, this makes the tasks ideal for testing inner speech models of AVHs.

### 1.3 Valence and externalising biases

A further question stemming from inner speech models of AVHs relates to what may cause one instance of inner speech to become misattributed, but not another instance. As discussed, source monitoring theories may appeal to vividness of mental imagery and the effort associated with cognitions, but theories of AVHs have also suggested that unpleasant, ego-dystonic cognitions are more likely to become misattributed (Morrison et al., 1995). This is consistent with reports that AVHs are more likely to occur in conditions of negative affect or stress (Nayani & David, 1996), and with the well-replicated finding that, at least in psychiatrically healthy individuals, negative events are more likely to be dismissed as attributable to a non-self cause (Mezulis, Abramson, Hyde, & Hankin, 2004).

However, the findings of studies that have investigated whether negatively valenced cognitions in source memory/signal detection tasks are more likely to become externally misattributed than neutral or positively-valenced cognitions have been inconsistent (Bendall et al., 2011, Morrison et al., 1995). In a recent meta-analysis of these studies (Brookwell et al., 2013), slightly larger externalising biases were observed in studies that employed positive (Hedges *g* = 0.75), than negative (*g* = 0.62), or than neutral stimuli (*g* = 0.50). However, because of the designs of the synthesized studies, it was not possible to examine whether these effect sizes differed from one another, which makes drawing any conclusions very difficult (especially when only five studies were included in the meta-analysis). Recent work investigating how emotion can modulate intentional binding (a low-level measure of sensorimotor agency) showed that intentional binding was reduced (i.e., participants’ sense of agency over their actions was reduced) when participants’ actions were paired with a negative emotional outcome in comparison to when actions were paired with a neutral or positive outcome (Yoshie & Haggard, 2013). Thus, there are reasons to believe that negatively-valenced cognitions are more likely to be misattributed to an external source than are neutral or positively-valenced cognitions. However, further work is needed to establish whether this is the case.

### 1.4 The present study

The present study therefore set out to test whether performance on an auditory signal detection task was affected by the generation of auditory verbal mental imagery, and whether this was modulated by the emotional valence of the stimuli, and/or self-reported hallucination-proneness, in a non-clinical sample. This extends previous research by directly linking the putative ‘raw material’ of AVHs – auditory imagery – with a frequently used task that can elicit false perceptions, with the participant engaged immediately in a decision about the perceptual origin of a stimulus.

Two key changes were made to previous paradigms investigating AVI and signal detection. Firstly, trials in which no signal was present were included, to allow the generation of ‘false alarm’ responses and the calculation of relevant signal detection measures. Secondly, the signal detected was a voice (as opposed to a pure tone), in order to maximise the relevance of the task to inner speech models of AVHs. We conducted two experiments which manipulated or measured the extent to which participants generated AVI. In Experiment 1, participants completed two blocks of auditory signal detection: one in which they were cued with a short sentence and required to generate AVI of that sentence whilst performing the task, and one in which there was no cued sentence or instruction to use AVI. In Experiment 2, a different sample of participants completed two blocks of auditory signal detection: one in which there was no cued sentence or instruction to use AVI, and one in which they were cued with a short sentence, but with no instruction to use AVI. Participants then retrospectively reported the extent to which they engaged in AVI whilst attempting to detect the auditory stimulus.

We predicted that use of AVI would lead to a lower response bias, because participants would have more opportunity to misattribute a self-generated event to an external source. Such a bias would lead participants to correctly detect more voices in the noise, but also incorrectly detect more voices in the noise (i.e., report hearing a voice when none was present). Furthermore, drawing on the previous literature linking signal detection performance and AVHs, we predicted that participants who scored highly on self-report measures of proneness to hallucinations may be particularly vulnerable to this effect. Finally, consistent with the findings of Yoshie and Haggard (2013), and with previous findings relating to self-attribution biases mentioned above, we predicted that participants’ response biases would be lower when they generated negative AVI than when they generated positive AVI.

## 2 Experiment 1

In the first experiment, participants performed an auditory signal detection task under two conditions: (1) with a visually presented verbal cue and an instruction to use AVI, and (2) with no cue, and no instruction to use AVI. We predicted that using imagery would lower participants’ response bias (making them more likely to report hearing a voice in the noise), but have no effect on sensitivity (the ability to distinguish between the voice and the noise). In half of the trials, participants were asked to engage in positive AVI (e.g., “I am good”) and in half, participants were asked to engage in negative AVI (“I am bad”). We predicted that participants’ response bias would be lower when generating negative AVI than when generating positive AVI.

### 2.1 Materials and methods

#### 2.1.1 Participants

The sample consisted of 125 participants from the undergraduate and staff population of Durham University, UK. Five participants were excluded from the final sample, due to technical malfunctions during the testing session (*n* = 3), or because their task sensitivity (*d*′ – see below) was classified as an outlier (>4.5 standard deviations above the mean) (*n* = 2). The final sample size was therefore 120 (number of females = 91; mean age = 20.7 years, *SD* = 2.5, range = 18–30 years). All participants were native English speakers and none reported any hearing problems. In return for their time, participants were given course credit or a small payment.

#### 2.1.2 Signal detection task

The task required participants to listen to bursts of noise, and to respond whether a voice stimulus was present in the noise. All participants completed two conditions: one block in which they were asked to use auditory verbal imagery whilst detecting the stimuli (the ‘AVI’ condition) and one block in which they were not (the ‘non-AVI’ condition).

**Fig. 1:**
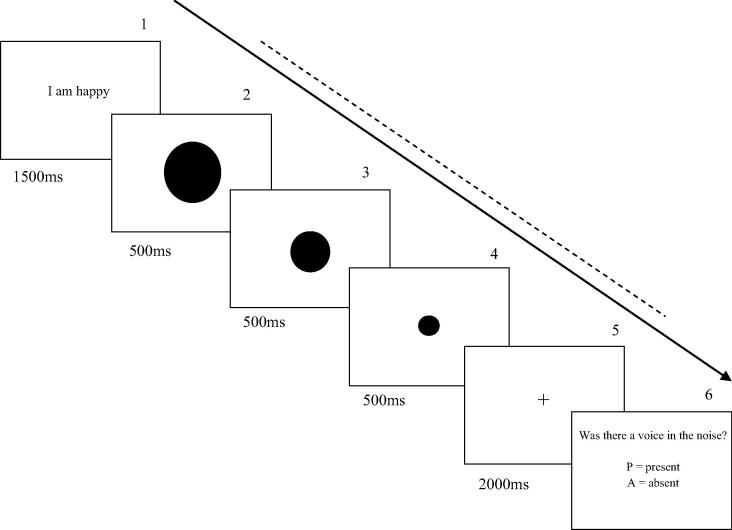
Schematic representation of a single trial in the AVI condition of the signal detection task. A sentence is presented to the participant (Screen 1), followed by a 1500 ms countdown (Screens 2–4), followed by a fixation cross, which, on voice-present trials, was accompanied by a voice stimulus (Screen 5). Participants were instructed to ‘imagine saying’ the presented sentence when they saw the fixation cross, and then provide a response as to whether they believed a voice was present during Screen 6. The proportion of the trial during which pink noise played is indicated by the dashed line.

Each block of the signal detection task (SDT) consisted of 80 trials, each lasting 5 s (plus response time). Fig. 1 shows an illustration of a single trial of the task. In the AVI condition, participants were first presented with a 3–4 syllable sentence, in the centre of the screen, for 1.5 s. In the non-AVI condition, participants were simply presented with a blank screen for 1.5 s. This was followed by an on-screen countdown, which consisted of a shrinking circle, to mimic a ‘3, 2, 1…’ countdown. (It was not appropriate to use a verbal on-screen countdown, as this may have interfered with processing of the presented sentence.) Pink noise (which consists of equal energy per octave; generated using Audacity 2.0.2) began playing through the provided headphones, simultaneously with the countdown. The countdown was followed by a fixation cross, which was present on the screen for 2 s. The participants were informed that, if there was a voice present in the noise, it would only appear when the fixation cross was present. Participants were then prompted to respond, with a button press, as to whether they believed a voice was present in the noise.

The sentence presented to the participant always took the form of the words ‘I am’, followed by an adjective (i.e., the participant was required to repeat a sentence about themselves). Half of the trials presented a positively valenced sentence (e.g., “I am happy”), whereas the other half presented negatively valenced sentences (e.g., “I am sad”). These sentences were rated for valence on a Likert scale (1 = very negative; 7 = very positive), by a separate subset of participants (*N* = 13), none of whom participated in the main experiments. The ratings of the words used for positive sentences (*M* = 6.04, *SD* = 0.42) were significantly higher than the words used for negative sentences (*M* = 2.13, *SD* = 0.66), *t*(65.9) = 31.53, *p* < .001 (equal variances not assumed). None of the mean ratings for any individual positive words were rated lower than any of the negative words. Positively and negatively valenced sentences were also matched for number of syllables.

In 44/80 trials, a voice stimulus was embedded in the pink noise as the fixation cross appeared on the screen, lasting for 1.5 s (‘voice-present trials’). The stimuli in the voice-present trials always spoke the same words that had been presented to the participant, in a male voice. The pink noise remained at the same volume for all trials for all participants; that is, only the volume of the voice stimuli differed between trials to vary the signal-to-noise ratio (voice stimulus to pink noise volume ratio). The signal-to-noise ratio in the trials varied between four different levels, based on the performance of pilot participants (*N* = 10, none of whom participated in the main experiment). Of the 44 voice-present trials in each signal detection block, 8 consisted of stimuli that pilot participants detected on 100% of trials, designed to ensure that all participants in the main experiment could correctly detect some of the voice stimuli. The remaining 36 trials were split evenly between volumes at which pilot participants detected the voice stimuli on 75%, 50% and 25% of trials. This was designed to maximise the ambiguity of the presented stimuli. In the remaining 36/80 trials, no voice was embedded in the noise (‘voice-absent trials’). The 44:36 ratio of present:absent trials was roughly based on previous auditory signal detection studies (e.g., Barkus, Stirling, Hopkins, McKie, & Lewis, 2007). Signal-to-noise ratio and the presence/absence of a voice stimulus were balanced across valence of the presented sentence.

#### 2.1.3 Measure of hallucination-proneness and AVI-usage

The Revised Launay–Slade Hallucination Scale (LSHS-R) was used to assess hallucination-proneness. This 9-item measure was adapted by McCarthy-Jones and Fernyhough (2011) from the 20-item Launay–Slade Hallucination Scale used by Morrison, Wells, and Nothard (2000). The scale assesses proneness to hallucinatory experiences in both the auditory and visual modality. It has been shown to have high internal reliability and a more valid factor structure than previous versions of the Launay–Slade Hallucination Scale.

Participants were also asked to estimate the extent to which they had generated AVI at the appropriate time during the task, giving a number between 0 and 100.

#### 2.1.4 Procedure

Participants wore over-the-ear headphones (Logik LHHIFI10) to complete the task. They were informed that they would be listening to bursts of noise, and listening out for a voice in the noise, responding present/absent with a button press at the end of each trial. All participants were told that some voices would be easier to hear, whereas others would be quieter and harder to detect, although they were not informed how often a voice was likely to be present. For the AVI condition, participants were instructed to “imagine saying the sentence to yourself silently”, at the same time as the fixation cross appeared on the screen. Participants were told they did not need to deliberately move their mouth or speak aloud during the main task, but simply to use auditory imagery of the presented sentence. They were also informed that, if there was a voice present in the noise, it would be presented at the same time as the fixation cross. During the practice phase of the AVI condition, participants were asked to speak the sentence out loud at the required time point for the first four trials, to ensure that they understood the instructions. If the participant did not vocalise the sentence at the appropriate time point, the practice trials were repeated until they were able to perform the task as requested. After the practice trials, participants were asked whether they understood the instructions relating to using AVI, and offered the chance to repeat the practice if unsure. In the event that participants did not understand the instructions relating to AVI (which was rare), various different descriptions were given to aid understanding (e.g., ‘inner speech’, ‘auditory imagery’ or ‘talking to yourself in your head’). In the non-AVI condition, participants were simply asked to detect a voice in the noise, but not given any instructions about imagining a voice.

All participants completed both conditions of the SDT. The order in which they completed the tasks was counterbalanced across participants. Between the two blocks of trials, participants completed the self-report items (see Section 2.1.2), and completed several other tasks (to be reported elsewhere).

#### 2.1.5 Data analysis

Performance on the SDT was analysed using signal detection theory. For each trial response, there were therefore four possible outcomes: hit (voice-present, ‘present’ response), miss (voice-present, ‘absent’ response), correct rejection (voice-absent, ‘absent’ response) and false alarm (voice-absent, ‘present’ response). From these, signal detection parameters relating to response bias (*β*) and sensitivity (*d*′) were calculated. Following Stanislaw and Todorov (1999), *β* was calculated as follows: $\beta =e\left\{\frac{Z{\left(\mathrm{FA}\right)}^{2}-Z{\left(H\right)}^{2}}{2}\right\}$. *β*, based on the likelihood ratio of the signal and noise distributions, was chosen as a measure of response bias to be consistent with previous studies relating auditory signal detection performance to hallucination-proneness (e.g., Varese et al., 2011, Vercammen et al., 2008) which have shown robust links to hallucination-proneness in a recent meta-analysis (Brookwell et al., 2013). A *β* value <1 corresponds to a bias towards responding ‘yes’, whilst a value >1 corresponds to a bias towards responding ‘no’. *d*′ is defined as the difference between the standardised hit rate and false alarm rate, with a value of 0 representing performance at chance level.

There were two within group variables: task condition and sentence valence. A median split was also performed on the data according to score on the LSHS-R, grouping the participants into high (scoring ⩾ 15 on the LSHS-R, *N* = 61) and low (scoring < 15, *N* = 59) hallucination-proneness; hence there was one between group variable: hallucination-proneness group (high/low). We therefore performed a 2 × 2 × 2 mixed model ANOVA, with response bias (*β*) as the dependent variable, and task condition (AVI/non-AVI), sentence valence (positive/negative) and hallucination-proneness group (high/low) as independent variables. This analysis was also repeated with sensitivity (*d*′) as the dependent variable, to test whether the manipulation affected participants’ ability to distinguish the voice from the noise. Where data was non-normally distributed, Mann–Whitney *U* tests were used during further analysis. Signal detection performance was also modelled using regression analysis to investigate the contribution of different variables to task performance in the different conditions.

### 2.2 Results

Participants generally reported being able to complete the task as instructed without difficulty, and reported using AVI with the fixation cross a relatively high amount (*M* = 91.7, *SD* = 9.0). There was no difference in the amount of AVI-usage reported between participants in the high (*M* = 90.58, *SD* = 9.2) and low (*M* = 92.88, *SD* = 8.6) hallucination-proneness groups: *t*(117) = 1.40, *p* = .16, *d* = 0.26. Descriptive statistics for response bias (*β*) and sensitivity (*d*′) to the auditory SDT under the two conditions (AVI/non-AVI) for high and low hallucination-prone participants, are shown in Table 1.

#### 2.2.1 Response bias (*β*)

**Fig. 2:**
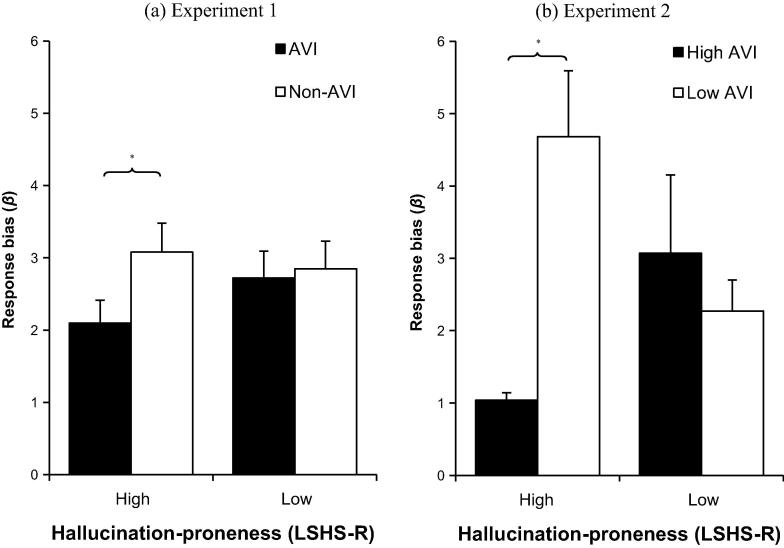
Response bias (*β*) in Experiment 1 & 2 auditory signal detection task. (a) Performance on the AVI and non-AVI condition, in high and low hallucination-prone participants. AVI = auditory verbal imagery; non-AVI = non-auditory verbal imagery. (b) Performance on the cued condition of the SDT, split by reported level of AVI-usage, and high and low hallucination-proneness. High AVI = participants who reported levels of AVI above the median; Low AVI = participants who reported levels of AVI below the median. Error bars = 1 SEM. ^∗^*p* < .01.

For descriptive statistics, see Table 1. For *β* (response bias), a 2 × 2 × 2 mixed model ANOVA showed a main effect of task condition (AVI/non-AVI): *F*_(1,118)_ = 5.99, *p* = .016, *η_p_*^2^ = .048, showing that participants performed with a lower response bias in the AVI condition (*M* = 2.41, *SD* = 2.7) compared to when not using AVI (*M* = 2.97, *SD* = 3.01). There was no overall effect of hallucination-proneness group (*F*_(1,118)_ = 0.43, *p* = .51, *η_p_*^2^ = .004). There was however, a significant task condition × hallucination-proneness interaction: *F*_(1,118)_ = 4.47, *p* = .037, *η_p_*^2^ = .037, (see Fig. 2a). Further analysis using Mann–Whitney *U* tests showed that the effect of AVI appeared to be specific to highly hallucination-prone participants, who had a significantly lower response bias in the AVI condition (*Mdn* = 1.12) than in the non-AVI condition (*Mdn* = 1.79): *z* = 3.51, *p* < .001, *r* = .45. In the low hallucination-prone participants, there was not a significant difference in *β* between the AVI condition (*Mdn* = 1.50) and the non-AVI condition (*Mdn* = 1.47): *z* = 0.11, *p* = .91, *r* = .01. There was no effect of sentence valence on *β*: *F*_(1,118)_ = 1.18, *p* = .28, *η_p_*^2^ = .01, nor any interactions between sentence valence and any other variables (all *p*s > .13).

Given that the use of median splits can sometimes result in spurious interaction effects (McClelland & Judd, 1993), we also analysed this data using two separate regression analyses. In one regression model, response bias (*β*) in the AVI condition was the dependent variable, with *β* in the non-AVI condition and Launay–Slade Hallucination Scale (hallucination-proneness) score (left as a continuous variable) as independent variables. This model significantly predicted *β* in the AVI condition (*F*_(2,119)_ = 23.3, *R*^2^ = .20, *p* < .001), with both response bias in the non-AVI condition (*β* = .41, *p* < .001) and hallucination-proneness (*β* = −.193, *p* = .02) emerging as independent predictors of response bias in the AVI condition. In the second regression model, response bias in the non-AVI condition was the dependent variable, with response bias in the AVI-condition and hallucination-proneness (again, left as a continuous variable) used as independent variables. This model significantly predicted response bias in the non-AVI condition (*F*_(1,119)_ = 11.62, *R*^2^ = .17, *p* < .001), although only AVI-condition response bias was a significant predictor (*β* = .41, *p* < .001), with hallucination-proneness not significantly predicting performance in this condition (*β* = .03, *p* = .74). Therefore, to summarise, when controlling for performance on the other signal detection condition, hallucination-proneness only significantly predicted response bias in the task condition in which participants used AVI.

Given that the to-be-detected voice was male, which could feasibly affect task performance between genders, the effect of gender on response bias was also investigated. There was no effect of gender on signal detection task performance (*F*_(1,116)_ = .12, *p* = .73, *η_p_*^2^ = .001), and gender did not interact with task condition (*F*_(1,116)_ < .001, *p* = .99, *η_p_*^2^ < .001), valence (*F*_(1,116)_ = .04, *p* = .84, *η_p_*^2^ < .001), or hallucination-proneness (*F*_(1,116)_ = .51, *p* = .73, *η_p_*^2^ = .001).

#### 2.2.2 Sensitivity (*d*′)

For descriptive statistics, see Table 1. For *d*′ (sensitivity), there was no effect of task condition (*F*_(1,118)_ = .148, *p* = .701, *η_p_*^2^ = .001) or hallucination-proneness (*F*_(1,118)_ = 2.09, *p* = .15, *η_p_*^2^ = .017), nor any interaction between task condition and hallucination-proneness (*F*_(1,118)_ = 2.39, *p* = .125, *η_p_*^2^ = .02). There was no effect of valence on *d*′ (*F*_(1,118)_ = .316, *p* = .575, *η_p_*^2^ = .003), nor any interactions between valence and any other variables (all *p*s > .23).

There was also no effect of gender on task sensitivity (*F*_(1,116)_ = .07, *p* = .79, *η_p_*^2^ = .001), nor any interaction between gender and task condition (*F*_(1,116)_ = .65, *p* = .42, *η_p_*^2^ = .006), valence (*F*_(1,116)_ = .05, *p* = .82, *η_p_*^2^ < .001), or hallucination-proneness (*F*_(1,116)_ = .40, *p* = .53, *η_p_*^2^ = .003).

### 2.3 Discussion

The first key finding from Experiment 1 was that when participants were instructed to use AVI during a signal detection task, there was a significant drop in response bias. That is, participants were more likely to respond that a voice was presented in the noise when they engaged in AVI, regardless of whether a voice was actually presented. However, using AVI did not affect participants’ sensitivity (ability to distinguish between the speech and the noise). Importantly, the interaction between task condition and hallucination-proneness indicated that the effect of AVI on response bias was specific to participants who scored highly on self-reported hallucination-proneness, whilst there was no difference between the imagery conditions in participants who reported few hallucinatory experiences. This finding suggests that the use of AVI caused hallucination-prone participants to exhibit a lower response bias. That is, when participants prone to hallucinations generate mental imagery, they are more likely to respond ‘present’, regardless of whether the stimulus was actually present or not. This is consistent with models that suggest AVHs may occur when inner speech is misattributed to an external source, which may link to excessively vivid mental imagery, low levels of cognitive effort, and/or aberrant predictive processes (Brookwell et al., 2013, Ford and Mathalon, 2005).

It could be argued that previous findings of lower response bias in signal detection tasks in hallucination-prone participants could be due to a higher rate of spontaneous AVI usage. This interpretation, though, is not supported by the present data, which indicated that there was no difference in AVI-usage between individuals scoring high and low in hallucination-proneness. However, since all participants were instructed to use AVI, this may have masked potential differences in spontaneous AVI usage (a possibility that is explored further in Experiment 2, below).

Contrary to our prediction, there was no effect of the emotional valence of the imagined sentence on performance, nor any interaction between valence and any other variables. This is consistent with previous research showing that, on a source memory task, the emotional valence of the stimuli did not affect performance or interact with hallucination-proneness (Bendall et al., 2011).

## 3 Experiment 2

Experiment 1 showed that using AVI during auditory signal detection led to a reduction in response bias, specifically in highly hallucination-prone individuals. However, one concern is that simply cuing participants with a sentence to imagine could have altered task performance, and that the observed effect could be due to priming of the sentence, rather than the use of AVI specifically (although the observed interaction with hallucination-proneness would still be of interest). The data from Experiment 1 is not capable of addressing this concern. Because almost all participants reported a high level of AVI use, it was not possible to determine whether the effect was specific to participants who engaged in high levels of AVI. To investigate this, we therefore conducted a second experiment, using identical stimuli, in which participants were not instructed to use AVI, but were still cued with a sentence before each signal detection trial. After task completion, participants were asked to report the extent to which they felt they had used AVI whilst performing the task. The rationale for this design was that it made it possible to investigate whether signal detection performance was associated with AVI use, even when participants were not explicitly instructed to use it. This design also tested whether participants that were more hallucination-prone reported using more spontaneous AVI, leading to a lower response bias, or whether the two interacted (i.e., participants who were both highly hallucination-prone *and* reported using high levels of AVI had lower response biases, but the overall level of AVI was not associated with hallucination-proneness). Based on the findings in Experiment 1, therefore, for Experiment 2 we predicted that there would only be a difference between the two task conditions in individuals scoring highly on hallucination-proneness *and* who reported using high levels of AVI whilst performing the SDT.

### 3.1 Materials and methods

#### 3.1.1 Participants

The sample consisted of 60 participants from the undergraduate and staff population of Durham University, UK, none of whom had taken part in Experiment 1 (number of females = 48; mean age = 19.73 years, *SD* = 2.5, range = 18–30 years). All participants were native English speakers and none reported any hearing problems. In return for their time, participants were given course credit or a small payment.

#### 3.1.2 Procedure

Using identical stimuli and equipment as in Experiment 1, participants completed two blocks of the SDT. Participants were given the same instructions for how to complete the task, with the only difference being that they were given no instructions relating to AVI. Therefore, they completed two conditions: a ‘non-cued’ condition (identical to the non-AVI condition in Experiment 1) and a ‘cued’ condition (in which the same sentences as in Experiment 1 were presented before each burst of noise, but there were no AVI instructions). Participants were informed that, in the cued condition, the sentence they were presented on-screen would be the same as the voice they were instructed to detect, although they were not required to be able to comprehend the sentence in the noise to respond ‘yes’.

As in the first experiment, participants completed the 9-item LSHS-R as a measure of hallucination-proneness (see Section 2.1.3) between the two blocks of the SDT. After completion of the tasks, participants were presented with the following question: ‘This question relates to the task in which you were presented with a sentence before listening to the noise. When the fixation cross appeared on the screen, did you find yourself using ‘inner speech’ to say the previously presented sentence? If yes, what percentage of the time do you think you did this? (0–100)’.

#### 3.1.3 Data analysis

As in Experiment 1, we performed a median split on the data according to LSHS-R score (high: ⩾15, *N* = 32; low: <15, *N* = 28). We also performed a median split on the data according to the amount of AVI reported by the participants (high: ⩾75, *N* = 32; low: <75, *N* = 28) and conducted a 2 × 2 × 2 mixed model ANOVA, with task condition (cued/non-cued) as a within-subject variable, and hallucination-proneness (high/low) and AVI use (high/low) as between-subject variables. Due to the lack of effect of valence in Experiment 1, we did not include valence as a within-subject variable in this experiment. The dependent variable was response bias (*β*) on the SDT. The analysis was also repeated using sensitivity (*d*′) as the dependent variable.

### 3.2 Results

Participants reported using AVI (‘inner speech’) a relatively high amount, considering that no instructions were given (*M* = 66.83, *SD* = 29.1), although estimates ranged from the bottom to the top of the scale (range = 0–100). A Mann–Whitney *U* test indicated that there was no difference between the high (*Mdn* = 77.50) and low (*Mdn* = 75.00) hallucination-proneness groups in the amount of AVI retrospectively reported (*U* = 436, *p* = .86, *r* = .02).

#### 3.2.1 Response bias (*β*)

For descriptive statistics, see Table 2. The 2 × 2 × 2 (task condition × AVI-usage × hallucination-proneness) mixed model ANOVA with *β* as the dependent variable indicated that there was no effect of task condition (*F*_(1,56)_ = .097, *p* = .76, *η_p_*^2^ = .002) on *β*. That is, the presentation of the to-be-detected sentence did not alter participants’ response biases. There was also no main effect of hallucination-proneness (*F*_(1,56)_ = .59, *p* = .45, *η_p_*^2^ = .01). There was, however, a main effect of reported AVI-usage (*F*_(1,56)_ = 4.46, *p* = .039, *η_p_*^2^ = .074), indicating that participants who reported high levels of AVI during the cued signal detection condition had a lower response bias (*M* = 2.09, *SD* = 2.22) than those who reported low levels of AVI (*M* = 3.55, *SD* = 2.92). There was no interaction between condition and hallucination-proneness (*F*_(1,56)_ = 2.17, *p* = .146, *η_p_*^2^ = .037). There was also no interaction between task condition and inner speech usage (*F*_(1,56)_ = .01, *p* = .91, *η_p_*^2^ < .001).

There was a significant interaction between hallucination-proneness and AVI-usage (*F*_(1,56)_ = 4.62, *p* = .036, *η_p_*^2^ = .076), although the three-way interaction between task condition, hallucination-proneness and inner speech usage was not significant (*F*_(1,56)_ = 3.16, *p* = .08, *η_p_*^2^ = .053). However, given that the interaction was close to significance, and that the AVI-usage variable specifically referred to the cued condition, we explored the result further by conducting two 2 × 2 [AVI-usage × hallucination-proneness] ANOVAs, for the cued and non-cued conditions separately.

For the non-cued condition, there was no effect of hallucination-proneness (*F*(_1,56)_ = 1.91, *p* = .17, *η_p_*^2^ = .033), no effect of AVI-usage (*F*_(1,56)_ = 2.36, *p* = .13, *η_p_*^2^ = .04) and no interaction between hallucination-proneness and AVI-usage (*F*_(1,56)_ = .44, *p* = .51, *η_p_*^2^ = .008). This is unsurprising, since the measure of AVI-usage specifically asked participants to estimate their usage of AVI only during the cued condition. For the cued condition, there was no main effect of hallucination-proneness (*F*_(1,56)_ = .07, *p* = .80, *η_p_*^2^ = .001), but there was a trend towards an effect of AVI-usage, with participants who reported high levels of AVI (*M* = 1.99, *SD* = 2.59) showing a lower response bias than those who reported low levels of AVI (*M* = 3.56, *SD* = 3.41), *F*_(1,56)_ = 3.75, *p* = .058, *d* = 0.52. Importantly, in the cued condition, there was a significant interaction between hallucination-proneness and AVI-usage (*F*_(1,56)_ = 9.12, *p* = .004, *η_p_*^2^ = .14). This interaction effect was explored by conducting two Mann–Whitney *U* tests for the cued condition, comparing *β* between the high/low AVI-usage groups for both the high and low hallucination-proneness groups. This showed that, for the low hallucination-proneness group, there was no significant difference in *β* between the high (*Mdn* = 1.60) and low (*Mdn* = 1.80) AVI-usage groups (*U* = 93.0, *p* = .84, *r* = .04). However, for the high hallucination-proneness group, there was a significant difference in *β* between participants who reported high levels (*Mdn* = 1.01) of AVI-usage and those who reported low-levels (*Mdn* = 2.53) of AVI-usage (*U* = 66.0, *p* = .02, *r* = .41).

Again, to further explore the data, we conducted a regression analysis using response bias (*β*) in the cued condition as the dependent variable. *β* in the non-cued condition, hallucination-proneness score, inner speech usage, and the interaction term (hallucination-proneness × inner speech usage) were included as independent variables. Although hallucination-proneness score was used as a continuous variable, inner speech usage was kept as a dichotomous variable because a large proportion of participants reported either very high or very low levels of inner speech (59.7% of participants reported using inner speech on either 0 or >80% of trials). This model significantly predicted response bias in the cued condition (*F*_(4,59)_ = 4.26, *R*^2^ = .24, *p* = .004.), with *β* in the non-cued condition (*β* = .32, *p* = .014) and hallucination-proneness (*β* = .81, *p* = .027) significantly predicting response bias. Inner speech usage was not a significant predictor (*β* = −.19, *p* = .116), although the inner speech usage × hallucination-proneness interaction did significantly predict response bias in the cued condition (*β* = .25, *p* = .033). When this analysis was repeated with response bias on the non-cued condition as the dependent variable, the model significantly predicted performance (*F*_(4,59)_ = 3.54, *R*^2^ = .21, *p* = .012), but only response bias on the cued condition emerged as a significant predictor (*β* = .33, *p* = .014), whereas hallucination-proneness (*β* = −.30, *p* = .43), inner speech usage (*β* = −.12, *p* = .33), and the interaction between the two (*β* = .05, *p* = .89), were not significant predictors. To summarise, the regression analysis indicated that, when controlling for the non-cued condition, hallucination-proneness, and inner speech usage, the interaction between hallucination-proneness and inner speech usage significantly predicted response bias on the cued condition. However, these variables did not predict response bias on the non-cued condition.

As in Experiment 1, there was no effect of gender on signal detection response bias (*F*_(1,53)_ = .10, *p* = .75, *η_p_*^2^ = .002), nor any interaction between gender and task condition (*F*_(1,53)_ = .29, *p* = .59, *η_p_*^2^ = .005), inner speech usage (*F*_(1,53)_ < .001, *p* = .99, *η_p_*^2^ < .001), or hallucination-proneness (*F*_(1,53)_ = .001, *p* = .98, *η_p_*^2^ < .001).

#### 3.2.2 Sensitivity (*d*′)

For descriptive statistics, see Table 2. A 2 × 2 × 2 (task condition × AVI-usage × hallucination-proneness) mixed model ANOVA with *d*′ as the dependent variable showed no significant effect of task condition (*F*_(1,56)_ = 1.98, *p* = .165, *η_p_*^2^ = .034), and no main effect of hallucination-proneness (*F*_(1,56)_ = 0.05, *p* = .82, *η_p_*^2^ = .001). In contrast to *β*, there was no main effect of AVI-usage (*F*_(1,56)_ = 1.52, *p* = .22, *η_p_*^2^ = .026), and no interaction between hallucination-proneness and AVI-usage (*F*_(1,56)_ = 2.57, *p* = .12, *η_p_*^2^ = .044). There was no interaction between task condition and hallucination-proneness (*F*_(1,56)_ = .254, *p* = .62, *η_p_*^2^ = .005). There was also no interaction between task condition and AVI-usage (*F*_(1,56)_ = .02, *p* = .90, *η_p_*^2^ < .001). However, as with response bias (*β*), there was an interaction, at the trend level, between task condition, hallucination-proneness and AVI-usage (*F*_(1,56)_ = 3.74, *p* = .058, *η_p_*^2^ = .044). As with *β*, two 2 × 2 [hallucination-proneness × AVI-usage] ANOVAs were conducted to explore this further. For the non-cued condition, there was no effect of hallucination-proneness (*F*_(1,56)_ = .18, *p* = .68, *η_p_*^2^ = .003), no effect of AVI-usage (*F*_(1,56)_ = 1.35, *p* = .25, *η_p_*^2^ = .024), nor any interaction between hallucination-proneness and AVI-usage (*F*_(1,56)_ = .36, *p* = .55, *η_p_*^2^ = .006). For the cued condition, there was no effect of hallucination-proneness (*F*_(1,56)_ < .01, *p* = .99, *η_p_*^2^ < .001), nor an effect of AVI-usage (*F*_(1,56)_ = 1.11, *p* = .30, *η_p_*^2^ = .019). However, for the cued condition, there was an interaction between hallucination-proneness and AVI-usage (*F*_(1,56)_ = 5.23, *p* = .03, *η_p_*^2^ = .085). Mann–Whitney *U* tests showed that, for participants in the low hallucination-proneness group, there was no difference in *d*′ between the high (*Mdn* = 1.23) and low (*Mdn* = 0.99) AVI-usage group (*U* = 78, *p* = .37, *r* = .17). For participants in the high hallucination-proneness group, those who were in the high AVI-usage (*Mdn* = 0.85) group had a significantly lower *d*′ score than those in the low AVI-usage group (*Mdn* = 1.37) (*U* = 57, *p* = .008, *r* = 0.47).

As before, we further explored this finding by conducting a regression analysis, this time with task sensitivity (*d*′) in each condition as the dependent variable. *d*′ in the non-cued condition, hallucination-proneness, inner speech usage, and the hallucination-proneness × inner speech usage interaction term were used as independent variables. Overall, the model significantly predicted *d*′ in the cued condition (*F*_(4,59)_ = 9.51, *R*^2^ = .41, *p* < .001). However, only *d*′ in the non-cued condition was a significant independent predictor (*β* = .62, *p* < .001). Hallucination-proneness (*β* = .36, *p* = .25), inner speech usage (*β* = −.05, *p* = .61), and the hallucination-proneness × inner speech usage interaction term (*β* = −.29, *p* = .36) were not significant predictors of *d*′ in the cued condition. The same pattern of results emerged when *d*′ in the non-cued condition was used as a dependent variable. *d*′ in the cued condition was a significant predictor of *d*′ in the non-cued condition (*β* = .62, *p* < .001), but hallucination-proneness (*β* = −.25, *p* = .44), inner speech usage (*β* = −.07, *p* = .54) and the interaction between the two (*β* = .15, *p* = .63) were not significant predictors of *d*′ in the non-cued condition.

As in Experiment 1, there was no effect of gender on signal detection task sensitivity (*F*_(1,53)_ = .04, *p* = .84, *η_p_*^2^ = .001), nor any interaction between gender and task condition (*F*_(1,53)_ = .08, *p* = .77, *η_p_*^2^ = .002), inner speech usage (*F*_(1,53)_ = .57, *p* = .45, *η_p_*^2^ = .011), or hallucination-proneness (*F*_(1,53)_ = 1.82, *p* = .18, *η_p_*^2^ = .033).

### 3.3 Discussion

Experiment 2 used identical auditory and visual stimuli as Experiment 1; only the task instructions differed, in that participants were not told that they should use AVI. After completing the task, participants estimated the extent to which they had spontaneously engaged in AVI after being cued with a sentence. Both response bias and sensitivity were affected by the presence of a sentence cue only in participants who both (a) reported high levels of hallucination-proneness *and* (b) reported using high levels of AVI whilst detecting a voice stimulus (although it should be noted that the three-way interactions only reached trend levels of significance). Nevertheless, these results indicate that if the sentence cue did not cause participants to use high levels of AVI, then it did not have an effect on task performance. However, within the group of participants who used high levels of AVI, only those participants who also reported high levels of hallucination-proneness showed a bias towards perceiving a voice in the noise, and showed reduced sensitivity, when cued with a sentence.

These results are partially consistent with the results from Experiment 1: they indicate that highly hallucination-prone individuals show a lower response bias when using AVI. However, unlike Experiment 1, the results from Experiment 2 indicated that use of AVI also affected sensitivity to the task in highly hallucination-prone individuals (although sensitivity was not predicted by hallucination-proneness in the regression analyses). This was an unexpected finding, which may be explained by a greater increase in the number of ‘false alarm’ responses relative to the increase in ‘hit’ responses. That is, if the participant mistook internal, self-generated AVI for an external, non-self-generated stimulus, it may have had a relatively smaller effect on the hit rate, especially if presentation of a stimulus at a low signal-to-noise ratio affected performance. For example, the presentation of voice stimuli (even below a participant’s auditory threshold) may have interfered with the likelihood that internally generated AVI was mistaken as external.

## 4 General discussion

To summarise, the two experiments reported in this paper examined the effect of the generation of auditory verbal imagery (AVI) on auditory signal detection, in participants who reported high or low levels of hallucination-proneness. Experiment 1 showed that, when instructed to use AVI, participants showed a lower response bias, being more willing to respond that a voice was present in noise (regardless of its actual presence), compared to performance on a standard auditory signal detection task. Further analysis showed that this effect was specific to participants who reported high levels of hallucination-proneness. Emotional valence of the material being imagined did not affect performance. Experiment 2 compared performance on a standard auditory signal detection task, and a variant of the task in which participants were cued with a sentence to detect, but not given any instructions to use AVI. The results suggested that hallucination-prone participants only showed a lower response bias when they retrospectively reported using AVI, despite not being instructed to do so. In Experiment 2, counter to expectations, there was some evidence that task sensitivity was also affected by usage of AVI.

These findings provide support for models of AVHs which suggest that they result from an external misattribution of an internal mental event, such as inner speech (Ditman and Kuperberg, 2005, Frith, 1992, Jones and Fernyhough, 2007b). The present studies partially support previous findings which have shown lower response biases in auditory signal detection, in both clinical and non-clinical samples that report frequent hallucinatory experiences (Brookwell et al., 2013), and extend the findings by showing that hallucination-prone individuals only showed a lower response bias when using AVI. As far as we are aware, previous studies that have linked performance on signal detection tasks to hallucinations have not incorporated variation in AVI/inner speech usage into their study design.

Given that, in both experiments, there was no association between level of reported AVI and level of reported hallucination-proneness, the results cannot be explained in terms of increased AVI usage in hallucination-prone individuals. Instead, the results suggest that when hallucination-prone individuals do use AVI, it is more liable to become externally misattributed. This is consistent with the previously outlined inner speech models of AVHs, suggesting that performance on reality discrimination signal detection tasks may be related to problems with self-monitoring of internally generated cognition. The present study does not, however, provide evidence to distinguish between precise mechanisms at play in reality discrimination or self-monitoring biases. It is possible that hallucination-prone participants misattributed AVI due to high levels of vividness of the imagery, making it harder to distinguish from a ‘real’ perception. High levels of vividness of mental imagery may be a trait shared by hallucination-prone individuals, which could lead to a higher likelihood of external misattributions. Alternatively, low levels of cognitive effort associated with AVI generation may have led to a similar effect in this group. Interestingly, hallucination-prone participants in the present study showed the opposite effect to that elicited by Perky (1910), instead showing patterns of response more consistent with those reported by Peterson and Graham (1974) or Farah and Smith (1983), who showed facilitation of perception by use of mental imagery. However, the present data support the hypothesis that this may be due to the effect of imagery on response biases, rather than sensitivity.

Neuroscientific findings describing activations in inner speech and those occurring during AVHs have implicated speech production areas, as well as primary and secondary auditory cortical regions, in the generation of AVHs (Allen, Larøi, McGuire, & Aleman, 2008), as well as showing higher levels of activity in auditory cortical (including speech perception) regions when patients with a diagnosis of schizophrenia use inner speech (Simons et al., 2010). Ford et al. (2001) previously showed that inner speech usage in hallucinating individuals was not associated with the same cortical attenuation in response to an external stimulus as in non-hallucinating individuals, suggesting that self-monitoring failures, which may underlie lack of agency over inner speech, are linked to aberrant predictive processes. To support this, a recent study showed that cortical attenuation to self-generated actions in individuals scoring highly on measures of schizotypy was reduced (Oestreich et al., 2015). Furthermore, Moseley, Fernyhough, and Ellison (2014) showed that modulating excitability in superior temporal regions affected performance on a signal detection task similar to the task used in the present study. In combination with the present results, this might imply that hallucination-prone individuals’ inner speech may be associated with higher levels of vividness, reflected in higher levels of activity in speech perception regions. This is supported by neuroimaging findings showing that auditory mental imagery (in a non-clinical sample) rated as higher in vividness is associated with higher levels of activity in speech perception regions (Zvyagintsev et al., 2013). This is also consistent with the conclusions of Aleman et al. (2003), who interpreted the effects of auditory imagery as evidence of higher perceptual detail in the AVI of hallucinators, and may suggest that earlier findings relating to the interaction between imagery and perception (Farah & Smith, 1983) are linked to reality discrimination biases through the perceptual detail involved in auditory imagery. These findings suggest that cortical attenuation in sensory regions, commonly linked to the sense of agency, may be linked to perceptual detail in mental imagery, and hence reality discrimination biases.

Unexpectedly, hallucination-prone participants did not show a lower response bias on signal detection overall: the effect was only observed when using AVI. In this respect, the results are inconsistent with previous findings (e.g., Barkus et al., 2007, Rankin and O’Carroll, 1995, Varese et al., 2011) which have suggested that hallucination-proneness is associated with a lower response bias in typical auditory signal detection. It is possible that task differences (for example, cueing the participants with a fixation cross at the point of the voice stimulus presentation, even in non-AVI versions of the task) could have affected performance in our study, and may make our results in non-AVI conditions non-comparable with previously conducted research. Interestingly, two studies which have previously used auditory signal detection paradigms in which participants were cued at the precise time point they should attempt to detect a voice, reported no association between hallucination-proneness and signal detection performance (Hoskin et al., 2014, Vercammen et al., 2008). It is possible that presentation of the cue with voice presentation may have focused attention on voice detection, and therefore reduced the rate of spontaneous AVI, which may have masked the association with hallucination-proneness. In future experiments, it would be informative to include a condition in which no voice presentation cue is included, to test this hypothesis. A further manipulation would be to engage participants in different types of mental imagery (e.g., visual or motor imagery) to test modality specificity of the effect of imagery on auditory signal detection. If only use of AVI elicited lower response biases in hallucination-prone individuals, this would provide further support for our interpretation that AVI could become misattributed to an external source (the noise). In contrast, if this effect was also elicited by visual imagery (on an auditory task), this would imply that more general processes may underlie the effect.

Indeed, one possible objection to the interpretation of this data as relating directly to the external misattribution of internal mental imagery relates to the role of working memory, and the cognitive load associated with generating AVI during the task. Research has previously shown that increasing working memory load can lead to a reduction in the sense of agency over self-generated actions (Hon, Poh, & Soon, 2013). From our data, it is not possible to rule out the possibility that the increased working memory load (by presenting a sentence to be detected) may have interacted with hallucination-proneness, which could underlie the observed effect. However, this explanation seems unlikely, given the relatively light cognitive load involved in our task. Hon et al., for example, did not find an effect of working memory on the sense of agency using a lower working memory load (two presented items), but did with a higher load (six presented items). This therefore seems like an unlikely explanation for our results.

It is also not possible to rule out that attentional processes may underlie the observed effect; for example, heightened attention to the to-be-detected stimuli in the AVI condition may have increased the participants’ willingness to respond that a voice was present (although the reverse could also be the case, in that heightened attention could plausibly decrease willingness to respond a voice was present). Contemporary cognitive theories have suggested that biased attentional processes may underlie some AVHs (Hugdahl et al., 2008), and it is likely that reality discrimination biases and attentional biases are not wholly independent constructs. Future research, though, should investigate the relation between working memory, attentional biases and auditory signal detection in relation to hallucinations.

A key area of research will be to understand what causes some instances of AVI/inner speech to become misattributed, but not others. The present study found no evidence that negatively valenced words were more likely to become misattributed, which does not provide evidence for the hypothesis that negative, ego-dystonic thoughts may be externalised and experienced as a hallucination (Morrison et al., 1995). This supports previous research using source memory tasks, which found that words associated with traumatic events were not more likely to be externally misattributed (Bendall et al., 2011), but stands in contrast to research suggesting that emotionally negative outcomes affect low-level sensorimotor agency (Yoshie & Haggard, 2013) and higher-level attributional biases (Mezulis et al., 2004). Previous research has, however, shown that inducing negative affect in participants causes an increase in the number of external misattributions on a typical auditory signal detection paradigm (Hoskin et al., 2014, Smailes et al., 2014). This might imply that the content of the inner speech does not play a role in its misattribution, but instead a general state of negative affect may cause an increase in the likelihood of external misattributions.

Furthermore, it is possible that the valence of the AVI may interact with individual schemas relating to the self-concept; that is, the likelihood that a negatively valenced statement will be externally misattributed may be related to the extent to which the individual holds negative views about themselves. It is possible that a negative self-concept would lead to fewer misattributions of negative items (as they may be more likely to be attributed to the self). If this were the case, emotional valence of the stimuli used in the signal detection task might be expected to interact with measures of self-esteem or negative schemas relating to the self, as opposed to proneness to hallucinations. Thus, future research should aim to examine the role played by positive and negative beliefs about the self, as well as the role of affective states, in modulating participants’ reality discrimination abilities. An alternative (although not exclusive) possibility is that dialogic inner speech (that takes on the quality of a back-and-forth conversation), or inner speech that includes the voices of other people, may be more likely to be misattributed under conditions of high cognitive load or stress (Fernyhough, 2004, Jones and Fernyhough, 2007a). Further research that manipulates qualitative aspects of AVI and investigates their interaction with affective state is merited.

## Figures and Tables

**Table 1:** Means and standard deviations for Experiment 1, showing performance on the auditory signal detection task in the AVI and non-AVI task conditions, for high and low hallucination-prone participants, for positively and negatively valenced stimuli (*M*, *SD*).

Hallucination-proneness	Valence	AVI		Non-AVI	
*β*	*d*′	*β*	*d*′
High	Positive	1.74 (1.5)	1.12 (0.6)	2.55 (1.8)	0.98 (0.6)
Negative	1.83 (1.6)	1.10 (0.7)	2.47 (1.8)	1.03 (0.5)
Low	Positive	2.37 (1.9)	0.92 (0.6)	2.36 (1.9)	0.93 (0.6)
Negative	2.15 (1.6)	0.90 (0.5)	2.17 (1.6)	1.00 (0.6)

AVI = auditory verbal imagery condition. Non-AVI = non-auditory verbal imagery condition. *β* = response criterion. *d*′ = task sensitivity.

**Table 2:** Means and standard deviations for Experiment 2, showing performance on the auditory signal detection task, for high and low hallucination-prone participants, who reported high and low levels of AVI (*M*, *SD*).

Hallucination-proneness	AVI-usage	Cued		Non-cued	
*β*	*d*′	*β*	*d*′
High	High	1.04 (0.4)	0.87 (0.6)	1.38 (0.7)	0.86 (0.6)
Low	4.68 (4.2)	1.40 (0.5)	3.25 (3.6)	1.14 (0.6)
Low	High	3.07 (3.5)	1.23 (0.6)	3.12 (3.7)	1.03 (0.5)
Low	2.27 (1.6)	1.04 (0.7)	3.87 (4.4)	1.11 (0.7)

AVI = auditory verbal imagery; *β* = response bias; *d*′ = sensitivity.

## Footnotes

1 Although meta-analytic evidence robustly shows that externalising biases are associated with hallucinations in clinical and non-clinical populations, it is, of course, possible that these are a result, rather than a cause, of experiencing hallucinations.

2 Farah and Smith (1983), however, note that many participants, when using auditory imagery of a pure tone, reported hearing the tone at times discordant with when the tone was actually presented, implying that imagery may have cause false detections. These false alarm responses were not quantified, however.
